# The Microbiota and Epigenetic Regulation of T Helper 17/Regulatory T Cells: In Search of a Balanced Immune System

**DOI:** 10.3389/fimmu.2017.00417

**Published:** 2017-04-10

**Authors:** Annie Luo, Steven T. Leach, Romain Barres, Luke B. Hesson, Michael C. Grimm, David Simar

**Affiliations:** ^1^St George and Sutherland Clinical School, University of New South Wales, Sydney, NSW, Australia; ^2^School of Women and Children’s Health, University of New South Wales, Sydney, NSW, Australia; ^3^The Novo Nordisk Foundation Center for Basic Metabolic Research, Faculty of Health and Medical Sciences, University of Copenhagen, Copenhagen, Denmark; ^4^Adult Cancer Program, Lowy Cancer Research Centre, Prince of Wales Clinical School, University of New South Wales, Sydney, NSW, Australia; ^5^Mechanisms of Disease and Translational Research, School of Medical Sciences, University of New South Wales, Sydney, NSW, Australia

**Keywords:** gut microbiota, epigenetics, regulatory T cell, T helper 17 cell, inflammatory bowel diseases, obesity, type 2 diabetes

## Abstract

Immune cells not only affect tissue homeostasis at the site of inflammation but also exert systemic effects contributing to multiple chronic conditions. Recent evidence clearly supports an altered T helper 17/regulatory T cell (Th17/Treg) balance leading to the development and progression of inflammatory diseases that not only affect the gastrointestinal tract but also have whole-body manifestations, including insulin resistance. Epigenetic mechanisms are amenable to both environmental and circulating factors and contribute to determining the T cell landscape. The recently identified participation of the gut microbiota in the remodeling of the epigenome of immune cells has triggered a paradigm shift in our understanding of the etiology of various inflammatory diseases and opened new paths toward therapeutic strategies. In this review, we provide an overview of the contribution of the Th17/Treg balance in the development and progression of inflammatory bowel diseases and metabolic diseases. We discuss the involvement of epigenetic mechanisms in the regulation of T cell function in the particular context of dysbiosis. Finally, we examine the potential for nutritional interventions affecting the gut microbiota to reshape the T cell epigenome and address the inflammatory component of various diseases.

## Introduction

**Figure 1:**
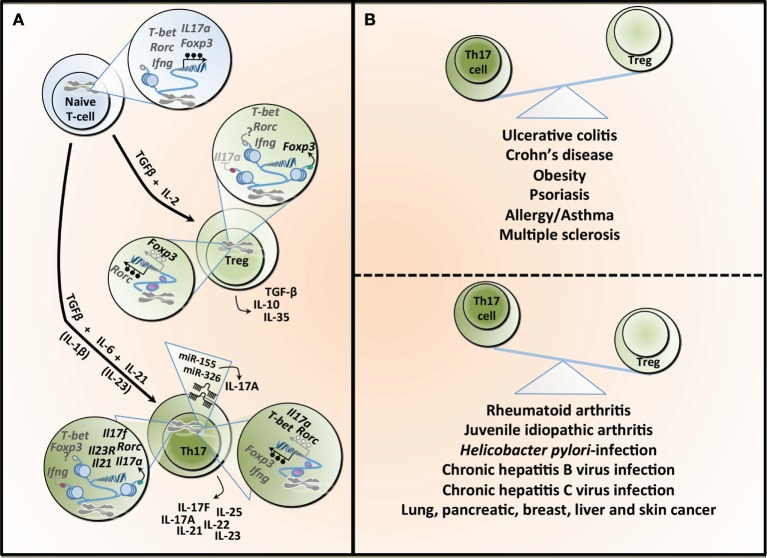
**T helper 17 (Th17) and regulatory T cell (Treg) lineage determination and the Th17/Treg balance in health and diseases**. **(A)** The differentiation of naïve T-cells into different lineages is regulated by specific cytokines. TGF-β in combination with IL-2 induces the expression of the transcription factor Foxp3 and Treg differentiation, which are characterized by the secretion of TGF-β, IL-10, and IL-33. TGF-β combined with IL-6 leads to the differentiation into Th17, which is characterized by the transcription factor Rorc (or RORγt). This process is amplified under the influence of IL-21 and both IL-1β and IL-23 have been reported to potentially contributing to Th17 differentiation. Th17 can secrete different cytokines, including IL-17A, IL-17F, IL-21, IL-22, IL-23, and IL-25. The differentiation into Treg or Th17 is a tightly controlled process regulated, at least partly, by epigenetic mechanisms. In naïve T-cell, the *T-bet* (or *Tbx1*) locus is characterized by both H3K4me3 (a permissive histone mark) and H3k27me3 (a repressive histone mark), whereas the *Rorc, interferon gamma* (*Ifng*), *Foxp3*, and *Il17a* loci show a total absence of such marks, making those five loci susceptible to both repression or expression of these genes. All five genes are hypermethylated in naïve T-cell contributing to gene silencing. Tregs show H3K4me3 on *Foxp3* and H3K27me3 on *Il17a*, supporting the expression of *Foxp3* while silencing *Il17a*. The demethylation of *Foxp3* and the hypermethylation of *Rorc* further contribute to *Foxp3* expression. In the same cells, *T-bet* and *Rorc* are marked by both histone modifications, whereas *Ifng* is devoid of these, rendering these loci more unstable. Th17 cells show H3K27me3 on *Ifng* and H3K4me3 on *Rorc, Il17a, Il17f, Il21*, and *Il23*, facilitating the expression of *Rorc* and supporting the secretion of the signature cytokines by these cells. This process is further supported by the hypermethylation observed in the *Ifng* and *Foxp3* loci, whereas the *Il17a, T-bet*, and *Rorc* are characterized by a demethylation, supporting gene expression. microRNAs can also contribute to the differentiation and function of Th17, and both miR-155 and miR-326 contribute to the production of IL-17A. TGF-β, transforming growth factor-beta; IL-2, interleukin-2; Foxp3, forkhead box P3; Rorc (or RORγt), retinoic acid receptor-related orphan receptor; T-bet, T-box1; H3K4me3, trimethylation of histone H3 on lysine 4 (green dot); H3K4me27, trimethylation of histone H3 on lysine 27 (red dot); IFN-γ, interferon gamma. **(B)** An imbalance between the number and activity of Th17 and Treg has been linked to several diseases. The predominance of Th17 is associated with inflammatory bowel diseases, obesity, and some allergic conditions, whereas Tregs have been linked to different forms of arthritis as well as several types of infections.

Immune homeostasis is a complex process involving a wide variety of key immunological players. Failure to establish a balanced immune response contributes to an ever-growing list of chronic diseases. Over the last few decades, the involvement of T helper 17 (Th17) cells and regulatory T cells (Tregs) in the maintenance of immune homeostasis has received increasing attention. It is now widely accepted that an altered Th17/Treg balance is associated with a wide variety of inflammatory diseases [Figure 1; (1–7)]. Given the role of epigenetic mechanisms in the regulation of immune functions, notably in the control of Th17 cell and Treg differentiation, it is not surprising that inflammatory diseases are linked to a substantial remodeling of the host epigenome in various tissues [Figure 1; (8–10)]. There is growing evidence that the gut microbial community is critical for the maintenance of a healthy host, and clear links between the gut microbiota and the central nervous system, or the cardiovascular and metabolic functions have been established (11–14). Consequently, perturbations of the gut microbiota (or dysbiosis), encompassing reduced microbial diversity and changes in microbiota composition, have been associated with a large number of chronic conditions (15). The vast majority of diseases that develop in the face of dysbiosis are characterized by immune alterations, and in particular chronic inflammation, which affect not only the gastrointestinal (GI) tract but also spread to the systemic circulation and central or peripheral tissues (16, 17). Some of the key manifestations of these inflammatory conditions, that include inflammatory bowel disease (IBD) and metabolic diseases, involve modulation of the local and circulating cytokine profile, but also significant changes in the frequency, function, and trafficking pattern of immune cells, including major alterations in the Th17/Treg balance, affecting the “immunological environment” in the host tissues (18–20). How dysbiosis orchestrates the remodeling of the T-cell landscape and whether this could involve epigenetic mechanisms still remain unclear. Perhaps an even more important question that remains unanswered is whether the gut microbiota could be manipulated to reshape the T-cell epigenome in the context of inflammatory diseases providing novel strategies to limit the pandemic of IBD and metabolic diseases. Although further work is still needed to provide definitive answers to those questions, recent progress in the field has shed light on important mechanisms driving the Th17/Treg balance and that could be targeted to treat inflammatory diseases.

## Th17/Treg Balance in IBDs

Th17 cells and Tregs are T-cell subsets that have essential roles in various immune processes, including inflammation, as well as disease progression. The Th17 lineage mediates effector functions and is under the transcriptional regulation of retinoic acid-related orphan receptor (ROR) γt (21–23). Th17 cells are further characterized by the production of their signature cytokines, including interleukin (IL)-17, IL-21, IL-22, IL-23, and IL-25, which promote tissue inflammation *via* the induction of other pro-inflammatory cytokines and chemokines [Figure 1; (23–25)]. At the other end of the immune regulation spectrum, natural Tregs and inducible Tregs differentiate in the thymus and periphery, respectively, and play an indispensable role in the suppressive control of both innate and adaptive immunity *in vivo* (26, 27). Developmentally, Tregs are regulated by the transcription factor forkhead box P3 (Foxp3) and express high levels of the IL-2-α chain receptor, CD25. Both Foxp3 and CD25 play pivotal roles in Treg differentiation, induction, and stabilization of their regulatory phenotype and immunosuppressive functions, including the secretion of IL-10, IL-35, and transforming growth factor (TGF)-β [Figure 1; (28–34)]. Tregs can be further divided into naturally occurring and peripherally induced Tregs. Phenotypically, these Treg cell subsets are indistinguishable from each other and utilize similar mechanisms to suppress immune responses including (1) cell-to-cell contact, (2) regulation of the effects of dendritic cells on effector cells, (3) secretion of inhibitory cytokines including IL-10, IL-35, and TGF-β, and (4) cytolysis of target cells (35). Interestingly, some studies have demonstrated that the development and function of a subset of peripherally derived Tregs are independent of Foxp3 expression and are characterized by their ability to produce high levels of the immunosuppressive IL-10 and TGF-β to suppress both adaptive and innate immune responses (36).

Despite these distinct functional differences, Th17 and Tregs are linked by their shared requirement for the cytokine TGF-β during development (21–23). This represents a close, fundamental relationship between the two cell types and further suggests shared developmental pathways during the progression of immune-mediated inflammatory conditions (22, 37). This is supported by data suggesting that the balance of Th17/Treg plays a role in the regulation of intestinal inflammation in IBD (38–40). A balance between the effector and regulatory functions (respectively mediated by Th17 cells and Tregs) is particularly important in the GI tract as it represents a critical barrier between the internal and external environments. The GI tract is under constant challenge from microbial and food antigens, which can generate both beneficial and harmful effects; hence, the normal intestinal immune system is constantly activated and tightly regulated. When this delicate balance between effector and regulatory responses is breached, the development of IBD ensues (38, 39). IBD are chronic inflammatory diseases of the GI tract, with unknown etiology, consisting of two major forms called ulcerative colitis (UC) and Crohn’s disease (CD), characterized by excessive inflammation of the intestine. The current hypothesis regarding the pathophysiology of IBD postulates that inflammation in these conditions is a result of the interaction of multiple factors including the intestinal microbiota, abnormalities of mucosal permeability, environmental and genetic factors, and a dysregulated host-immune response (41–44). Nonetheless, it still remains unclear whether chronic inflammation is responsible for the development of dysbiosis in IBD, or if disturbance in the gut microbiota is the trigger leading to inflammation in the GI tract. Despite the fact that animal models have suggested the possibility of dysbiosis preceding chronic inflammation (45), similar evidence is yet to be clearly demonstrated in human studies.

Changes in Th17 cells and Tregs numbers and functions are well established in both patients with IBD and murine colitis models. Elevated transcript levels of *IL17* have been reported in the mucosa of both UC and CD patients compared to healthy controls [Table 1; (2, 3)]. The relationship between IL-17 and IBD genesis, however, is likely to be complex, given that anti-IL-17 monoclonal antibody therapy failed to improve outcomes in CD (46). Serum levels of IL-22, another Th17-related cytokine, are significantly increased in IBD patients, an observation further recapitulated in a mouse model of colitis (47, 48). This excessive pro-inflammatory response results from a decreased differentiation and accumulation, as well as functional defects in Tregs in the inflamed tissues (49) or in peripheral blood of IBD patients with active disease (50). The upregulation of these pro-inflammatory cytokines is correlated with a significant reduction in the levels of several immunosuppressive cytokines including TGF-β, IL-10, and IL-33 (41, 49). All these events favor the establishment of a local effector immune response, leading to a disturbed intestinal immune system, resulting in chronic tissue injury. Accordingly, the modulation of the immunological balance between Th17 cells and Tregs could be exploited to revert the pro-inflammatory intestinal environment. Indeed, several studies have demonstrated that the re-tuning of the Th17/Treg balance could dampen down intestinal inflammation resulting in the re-establishment of intestinal immune homeostasis (40, 51). For instance, all-trans retinoic acid (ATRA) downregulated pro-inflammatory responses in both murine and human colitis by shifting the Th17/Treg profile (51). In this study, the upregulation of Foxp3 post treatment with ATRA and elevated levels of regulatory cytokines including IL-10 and TGF-β were associated with the downregulation of IL-17. Furthermore, metformin was able to attenuate IBD severity and reduce inflammation through the regulation of *Il17* and *Foxp3* expression, demonstrating the importance of the Th17/Treg balance in the regulation of intestinal inflammation [Table 1; (40)]. Taken together, these data support the notion that the attenuation of chronic intestinal inflammation in IBD is achieved through the restoration of a fine balance between pro- and anti-inflammatory responses (40, 51–54).

**Table 1:** **The T helper 17/regulatory T cell (Th17/Treg) balance in IBD and metabolic disease**.

Experimental model	Th17/Treg	Changes	Samples	Reference
**IBD**				
Patients with active UC and CD	*Il17* expression	↗	CD3^+^ T cells from colonic mucosa	(2)
Patients with UC and CD	*Il17* expression	↗	Mucosa	(3)
Patients with active CD	*Il22* expression	↗	Inflamed colonic lesions	(47)
Murine colitis	*Il22* expression	↗	Colon	
Patients with CD	IL-22	↗	Plasma	(48)
Murine colitis	*Il22* and *Il17* expression	↗	Inflamed colon	
IBD patients	Th17 and Th1/Th17 cells	↗	Lamina propria	(50)
	Tregs	↘	Peripheral blood	
IBD patients	IL-17A, IL-21, IL-23	↗	Inflamed mucosa	(41)
	TGF-β, IL-10, IL-33	↘	Inflamed mucosa	
**Metabolic diseases**				
Obese individuals	Tregs	↘	Omentum fat	(4)
Obese mice	Tregs	↘	Visceral adipose tissue (VAT)	
Obese patients with T2D	IL-17A, IL-17F, IL-21, IL-9	↗	Culture supernatant from activated PBMCs	(55)
Obese individuals	Tregs	↘	Adipose tissue	(58)
	Th17 cells	↗	Peripheral blood	
Obese individuals	Tregs	↘	Adipose tissue	(57)
Mice fed HFD	Th17^+^ CD4^+^ T cells	↗	Spleen	(56)
Mice fed HFD	% Foxp3^+^ CD4^+^ T cells	↘	VAT	(5)
*ob/ob* mice	% Foxp3^+^ CD4^+^ T cells	↘		
Mice immunized with ileum microbiota from mice fed HFD	IL-17^+^ and Foxp3^+^ CD4^+^ T cells	↘	Intestine	(1)

*IBDs, inflammatory bowel diseases; UC, ulcerative colitis; CD, Crohn’s disease; IL, interleukin; Tregs, regulatory T-cells; TGF-β, transforming growth factor beta; T2D, type 2 diabetes; PBMCs, peripheral blood mononuclear cells; HFD, high-fat diet; Foxp3, forkead box P3*.

*Red arrow pointing toward upper right: increased levels; black arrow pointing toward lower right: decreased levels*.

## Th17/Treg Balance in Metabolic Diseases

The Th17/Treg balance has also been linked with the development of obesity-associated diseases such as type 2 diabetes (T2D) [Table 2; (1, 4, 5, 7, 55–57)]. Immunologically, obesity is characterized by low-grade chronic inflammation with abnormal cytokine production and the activation of various inflammatory pathways, associated with adverse clinical outcomes including insulin resistance and T2D (58). In obesity, the polarization of a Th17 phenotype, especially in secondary lymphoid organs (e.g., spleen and lymph nodes), contributes to the development of other chronic inflammatory diseases, including experimental autoimmune encephalomyelitis (EAE) and asthma (58). Similarly, the severity of trinitrobenzenesulfonic acid-induced colitis in obese mice was found to be associated with Th17 expansion (58). Despite their pathogenic role in the initiation and maintenance of chronic inflammation, Th17 cells have also been implicated as critical players in the control of adipogenesis and glucose homeostasis in the context of obesity (19, 59). In a recent study, intestinal RORγt^+^ IL-17^+^ CD4^+^ T-cells were shown to participate in energy metabolism in mice. Indeed, a reduction in the numbers of RORγt^+^ and IL-17-producing CD4^+^ T-cells contributed to the development of insulin resistance (1). This observation supported the role of the intestinal Th17 lineage in the control of insulin sensitivity, an effect that differed from peripheral Th17 cells. Obesity also affects the numbers of Tregs and function in the regulation of whole-body metabolism. Several studies have demonstrated that visceral adipose tissues (VATs) of lean mice are highly enriched with a unique population of Foxp3^+^ CD4^+^ Tregs that possess a distinct T-cell receptor repertoire, pattern of chemokine and chemokine receptor expression, and gene expression profile compared to other tissues (4–7). Under obese conditions, the abundance of these VAT-resident Tregs is markedly reduced. Several studies have suggested that this depletion of Tregs plays a role in the inflammatory process that may contribute to obesity-associated diseases such as T2D (4–7). Foxp3^+^ VAT-resident Tregs express unusually high levels of the peroxisome proliferator-activator receptor (PPAR)-γ (6), a key nuclear receptor controlling adipogenesis that is also a target of the insulin-sensitizing drug thiazolidinediones (60). The functional importance of PPAR-γ expression for VAT-resident Tregs was further reinforced by the observation that Treg-specific deletion of PPAR-γ leads to a significant reduction in the number of Tregs in VAT, but not in other tissues (6). Collectively, these data highlight the role of PPAR-γ-expressing Foxp3^+^ Tregs in the control of inflammation induced by obesity and in metabolic diseases such as T2D (6).

**Table 2:** **Changes in gut microbiota in IBD and metabolic diseases**.

Experimental model	Microbiota involved	Changes	Samples	Reference
**IBD**				
Patients with UC and CD	*Bacteroidetes*	↘	Colon	(84)
	*Firmicutes, Lachnospiracea*	↘		
	*Proteobacteria*	↗		
	*Firmicutes, Bacillus*	↗		
	*Proteobacteria*	↗	Small intestine	
	*Firmicutes, Bacillus*	↘		
Patients with UC and CD	*Enterobacteriaceae*	↘	Colon	(85)
	*Bacteroidetes*	↘		
European children (at risk of IBD) vs children from Burkina Faso	*Bacteroidetes*	↘	Fecal samples	(87)
	*Firmicutes*	↗		
	*Enterobacteriaceae*	↗		
	Short-chain fatty acids (SCFAs)	↘		
Patients with UC	*Roseburia hominis*	↘	Fecal samples	(88)
	*Faecalibacterium prausnitzii*	↘		
	SCFAs	↘		
Patients with CD (vs UC and control)	*Proteobacteria*	↗	Site of active disease including ileum and colon	(89)
	*Bacteroidetes*	↗		
	*Clostridia*	↘		
Patients with UC	*Proteobacteria*	Unchanged	Patients with UC	
	*Bacteroidetes*	Unchanged		
	*Clostridia*	Unchanged		
Patients with CD	*Enterobacteriaceae*	↗	Fecal samples	(86)
	*Coccoides*	↘		
**Metabolic diseases**				
Obese individuals	*Bacteroidetes*	↘	Fecal samples	(109)
	*Firmicutes*	↗		
	*Actinobacteria*	↗		
Obese individuals	*Bacteroidetes*	↘	Fecal samples	(108)
	*Firmicutes*	↗		
Obese/overweight individuals	*Bacteroidetes*	↗	Fecal samples	(111)
	*Firmicutes*	↘		
	*Firmicutes/Bacteroidetes*	↘		
	SCFAs	↗		
Patients with T2D	*Firmicutes*	↘	Fecal samples	(112)
	*Clostridia*	↘		
	*Betaproteobacteria*	↗		
*ob/ob* mice	*Bacteroidetes*	↘	Cecal samples	(106)
	*Firmicutes*	↗		
	*Firmicutes/Bacteroidetes*	↗		
HFD fed mice	*Bacteroides MIB*	↘	Cecal samples	(114)
	*E. rectale–C. coccoides*	↘		
	*Bifidobacterium* spp.	↘		
	*Enterobacteriaceae*	↘		

*IBD, inflammatory bowel diseases; UC, ulcerative colitis; CD, Crohn’s disease; T2D, type 2 diabetes; HFD, high-fat diet*.

*Red arrow pointing toward upper right: increased levels; black arrow pointing toward lower right: decreased levels*.

## Epigenetic Regulation of the Th17/Treg Balance

The differentiation of Th17 cells and Tregs from naïve CD4^+^ T-cells is not only controlled by a combination of their cytokine milieu and transcriptional activities but is also subjected to epigenetic control through various mechanisms including DNA methylation (10, 61–63), changes in microRNA (miRNA) expression (64–69), and histone modification (22, 70–74), illustrating the complexity of CD4^+^ T-cell lineage decisions (Figure 1).

It has been proposed that the induction, maintenance, and function of Foxp3 are regulated by epigenetic mechanisms (9, 10, 75). A seminal study revealed the complete demethylation in a region within the evolutionally conserved non-coding sequence elements of the *Foxp3* gene and termed this region as the Treg-specific demethylation region (TSDR) (61). It was reported that demethylated TSDR is strongly associated with the stable expression of *Foxp3* in Tregs (61) and germ-line deletion of the TSDR resulted in the loss of *Foxp3* expression (10). However, there is evidence suggesting that TSDR demethylation is not an on or off switch but instead determines the stability of *Foxp3* expression (61–63). Interestingly, demethylation of the TSDR with DNA demethylating compounds is associated with enhanced immunosuppressive function of Foxp3^+^ Tregs and prevents the occurrence of diabetes in mice (75). Collectively, these data established that the demethylated TSDR contributes to the stable expression of Foxp3 in Tregs.

microRNAs are emerging as critical regulators of Th17 and Treg cell differentiation and function and consequently are also implicated in a number of diseases including IBD, diabetes, neurological diseases, and cardiovascular diseases (64, 65). Several studies have demonstrated that miR-155 is a prominent regulator of both Th17 and Treg differentiation and function *in vitro* and *in vivo* (64–69). For instance, miR155 is required for Treg development as miR-155-deficient (miR-155^−/−^) mice show a marked decrease in the frequency and absolute numbers of Tregs in both the thymus and secondary lymphoid organs (68). Despite a reduced number of Tregs, miR-155 is dispensable for Treg immunosuppressive function. This was confirmed by the finding that miR-155^−/−^ mice did not spontaneously develop GI diseases, as seen in patients with IBD. Moreover, Tregs from miR-155^−/−^ mice retained the capacity to prevent colitis induced by the adoptive transfer of CD4^+^ CD45RB^high^ T-cells into lymphopenic hosts (68). The above findings were not only recapitulated, but miR-155 was also found to be crucial in directing Th17 differentiation and enhancing IL-17A secretion by Th17 cells by directly inhibiting the SOCS1 (suppressor of cytokines signaling) signaling pathway (66). It also positively regulates Th17 cell function, but not that of Tregs (66). Together, these studies revealed that miR-155 is a critical driver of both Th17 and Treg development. Other miRNAs have also been reported to control Th17 and Treg development and function. For example, miR-182, miR-10a, and miR-17-92 are key regulators of Treg specification, stability, and their suppressor function in infectious and autoimmune diseases (76–78). miR-21 promotes Th17 differentiation and mediates the development of EAE as shown by the association between the defect in Th17 differentiation and the strong resistance to EAE in miR-21-deficient mice (79). In addition, anti-miR-21 treatments drastically reduced Th17 cell numbers as well as the severity of EAE (79). Likewise, miR-326 also drives the differentiation as well as migration of Th17 cells to inflammatory sites and the production of IL-17A which all potentiate the development of EAE (80).

Evidence from genome-wide epigenetic analysis of Th17 cells uncovered the enrichment of active histone modifications such as histone H3 acetylation and trimethyl-histone H3 lysine 4 (H3K4me3) in the promoters of cytokine and lineage-specific genes such as *IL17a, IL17f, IL21, IL23R*, and *ROR*γ*t* (22, 70–72). This suggests histone modifications such as acetylation or methylation play a role in Th17 cell lineage commitment (22). Similarly, promoters of Treg-related genes, such as *Foxp3*, are also marked with the permissive H3K4me3 modification, which correlates strongly with gene expression (72). Furthermore, DNA demethylation of the TSDR was reported to correlate with increased levels of H3K4me3, suggesting that specific DNA demethylation and histone modifications have similar roles in the determination and commitment of the Treg lineage (73, 74). More recently, two histone acetyltransferases, CREB-binding protein (CBP) and p300, have been shown to control Treg suppressive function and stability (81). A combined Treg-specific deletion of *CBP* and *p300* led to rapid development of fatal autoimmunity in mice within 3–4 weeks of birth. This was accompanied by decreased expression of genes characteristic of the Treg lineage, presence of autoantibodies, and increased infiltration of mononuclear cells in multiple organs (81), which resembles the clinical phenotype of scurfy or other *Foxp3* mutant mice (82). Supporting the observation that Tregs are prone to lose *Foxp3* expression in inflammatory diseases, CBP, and p300 act on the TSDR to control Treg stability and function during inflammation (81). These findings not only provide insight into the importance of histone acetyltransferases in the regulation of Treg development and function but also delineate distinct roles for CBP and p300 in the control of Tregs (81).

## Dysbiosis and the Th17/Treg Balance in IBD and Metabolic Diseases

As illustrated above, the Th17/Treg balance plays a critical role in control of the inflammatory process in IBD (38, 39). Although the exact etiology of IBD remains elusive, it is well accepted that this group of diseases preferentially affects genetically susceptible individuals and could result from a disproportionate activation of the immune system in response to changes affecting the gut microbiota [Tables 1 and 2; (44)]. Indeed, IBD is characterized by significant disturbances in the gut microbiota (83) and alterations in the balance between the dominant bacterial groups, depletion of particular bacteria, as well as a reduction in the diversity of the gut microbiota (84–86). A seminal study in the field recently identified that European children, considered to be at increased risk of IBD, were characterized by a reduced diversity in their gut microbiota and a total depletion of bacteria responsible for the digestion of dietary fibers when compared to their African counterparts (87). In this particular study, striking differences were observed in the diet these two groups of children were exposed to, highlighting the importance of nutritional habits in the maintenance of a healthy gut microbiota and the dramatic consequences of unfavorable dietary habits. Consistent with this observation, several studies have reported dramatic alterations in the proportion of different groups of bacteria in the context of IBD. A relative depletion of members of *Bacteroidetes* and *Lachnospiraceae* (84) or a decrease in *Roseburia hominis* and *Faecalibacterium prausnitzii* (88) has been observed in patients diagnosed with CD or UC. However, contradictory results have also been observed, with patients with IBD showing an increase in both *Proteobacteria* and *Bacteroidetes* (89) or in *Enterobacteriaceae* (86). This discrepancy could be the result of the variability in the type of biological samples being investigated, ranging from biopsy samples to fecal samples, or even the sites where such biopsies are taken from in the GI tract [Table 2; (90)]. Despite the inherent challenges of identifying key groups of bacteria that contribute to the development of IBD, dysbiosis is thought to be heavily involved in the pathophysiology of the disease. In particular, the ensuing immune activation and the establishment of chronic inflammation in the GI tract represent key steps in the development of IBD. Clear associations have now been established between the microbiota and T-cell differentiation or polarization and could thus link dysbiosis to changes affecting the Th17/Treg balance in IBD (91). The contribution of both dysbiosis and immune disturbance to IBD is further supported by the efficacy of anti-inflammatory, immunosuppressant, and immunomodulatory drugs, as well as microbiome modulators in the therapeutic arsenal for IBD (92, 93). In recent years, there has been a growing interest in the use of probiotics and prebiotics to treat or manage IBD, through the correction of intestinal dysbiosis (93). However, conflicting results have been reported regarding the use of probiotics or prebiotics to treat IBD, and both their efficacy and safety still remain to be established. For instance, promising results have been reported regarding the use of the probiotic, *Escherichia coli* Nissle 1917, to maintain remission in UC (94, 95). However, evidences supporting its use in the context of CD are still lacking (96). Some studies even reported that probiotics could cause harm (93). Similarly, if the beneficial effects of prebiotics in CD and UC remain elusive, some randomized studies using placebo as control have shown an improvement in GI symptoms and side effects following prebiotics supplementation in CD (97, 98). Another approach that has received much attention is the therapeutic manipulation of the intestinal microbiota *via* fecal microbial transplantation (FMT) to restore symbiosis in IBD patients (99). If some studies have reported results that could suggest some potential benefits of FMT in UC (100, 101), significant concerns remain regarding safety. In particular, screening systems to identify the nature of the stool that is likely to be safe for donation and might provide some benefits are still lacking. Indeed, adverse effects of transplanting diseased fecal microbiota have been demonstrated (102). Impaired intestinal function, including increased intestinal barrier dysfunction, faster GI transit, and innate immune activation, as well as exhibition of anxiety-like behavior, has been observed in mice that received fecal microbiota from patients with irritable bowel syndrome (102). These results suggest that the use of FMT as a management approach in IBD requires careful consideration and further research is warranted to establish its safety.

If chronic inflammation and alterations of the Th17/Treg balance are an integral component of IBD, a wide range of diseases increasingly recognized as comprising chronic inflammation, including obesity and T2D, may also be associated with such alterations. Reports supporting similarities in the physiopathology of IBD and metabolic diseases have recently started to emerge and have identified dysbiosis as a common trait shared between these conditions (83, 103). Indeed, changes in the microbiota that accompany alterations in nutritional habits and the development of obesity have been identified as critical contributors to the development of metabolic complications including insulin resistance and T2D and could even precede the establishment of local and systemic inflammation (83, 103, 104). Similar to what has been reported in IBD, both a decline in diversity and a remodeling of the bacterial component of the microbiota have been observed in the early stages of the development of obesity and insulin resistance (105). In mice, obesity has been linked to a decrease in *Bacteroidetes* and an increase in *Firmicutes*, as well as an increase in the *Firmicutes*/*Bacteroidetes* ratio (106). Similar changes have also been observed in mice fed a Western diet and these alterations could even be transmitted to germ-free mice by colonization using fecal microbiota obtained from obese mice (107). These alterations in the gut microbiota in response to obesity have since been confirmed in obese individuals or patients with T2D (108–110), although conflicting results have also been reported (111, 112). It is noteworthy that changes affecting the microbial flora associated with obesity and insulin resistance have also been shown to be reversible in response to treatment using prebiotics or probiotics (113, 114). Indeed, mice fed a high-fat diet (HFD) and supplemented with the prebiotic oligofructose showed increased *Bifidobacterium* spp., improved glucose metabolism, and reduced plasma and adipose tissue pro-inflammatory cytokines (114). Similarly, supplementing mice fed a HFD with the probiotic bacteria *Lactobacillus paracasei* ssp. paracasei F19 significantly reduced fat accumulation (113). Such beneficial effects were further confirmed in a clinical trial where adults with obese tendencies, who received the probiotic *Lactobacillus gasseri* SBT2055, were characterized by improved body composition and in particular decreased visceral and subcutaneous fat (115). These changes in the adipose tissue were associated with a decreased expression of pro-inflammatory genes in obese mice (116). These alteration were mediated by the regulation of macrophage infiltration and their regulatory rather than inflammatory polarization in the adipose tissue (117), confirming the effect of the gut microbiota on inflammation in metabolic diseases.

Although it is clear that a link exists between the gut microbiota and the immune system, this association is complex. While the gut microbiota can contribute to shaping or even to activating the immune system, the local immune environment can also remodel the gut microbiota (91, 118). In lean and healthy individuals, the gut microbiota and the intestinal immune system are in homeostasis, preventing bacteria and bacterial products leaking from the gut lumen to the underlying mucosa. This is partly achieved through the secretion of immunoglobulin A and the production of mucin by goblet cells. In this context, an anti-inflammatory environment is maintained through the secretion of TGF-β, as well as IL-25 and IL-33 by intestinal epithelial cells (IECs), favoring Tregs (119). In the face of dysbiosis, this fragile equilibrium is disrupted, which leads to immune activation. Both a reduction in microbiota diversity and an altered composition of the microbiota are associated with decreased mucin production and increased intestinal permeability. Increased translocation and exposure of the underlying mucosa to pathogenic bacteria and bacterial products trigger the activation of the inflammasome, and increases toll-like receptor (TLR) and NOD-like receptor signaling, resulting in the production of IL-1β, IL-6, IL-12, IL-18, and IL-23 (119). This pro-inflammatory milieu can lead to the activation of the adaptive immune system and shift the Th17/Treg balance. Although both the gut microbiota and the immune system are involved in the pathogenesis of IBD, it remains unclear which may be the initial disease trigger. As described above, dysbiosis can lead to both intestinal and systemic inflammation, but the immune system may also shape the gut microbiota (118). In the context of IBD where genetic susceptibility (e.g., genetic deficiency in TLR1) and/or environmental factors (pathogens) are present, immune activation and the resulting inflammatory process could contribute to altering IECs function and affect their secretory pattern, resulting in a remodeling of the gut microbiota (120).

The relationship between the gut microbiota and the immune system, and in particular the Th17/Treg balance, is now well established. Although it remains unclear as to which may be driving inflammatory diseases characterized by dysbiosis, their tight interaction contributes to the vicious circle of chronic intestinal inflammation.

## Microbiota-Derived Metabolites and the Regulation of Th17/Treg Cells

It has been clearly established that the commensal microbial community can influence the Th17/Treg balance, as supported by the remodeling of that balance in germ-free mice or in mice treated with antibiotics (121–124). Although the exact mechanisms are still unclear, factors and metabolites derived from the gut microbiota have received significant attention. *Bacteroides fragilis* has been reported to protect against experimental colitis through the release of polysaccharide A [PSA; (125)]. This anti-inflammatory effect was mediated by a decreased production of IL-17 by immune cells in the intestine and through the promotion of CD4^+^ T-cells differentiation to IL-10-producing cells and Foxp3^+^ Tregs (126). Similarly, *Bacteroides thetaiotaomicron* has been reported to exert anti-inflammatory effects by regulating the nuclear-cytoplasmic shuttling of PPAR-γ in both epithelial and intestinal cell lines (127). Although the effect on T-cells was not tested in that study, considering the critical role played by PPAR-γ in the differentiation of Treg, these findings confirm that the gut microbiota has a strong potential to influence the T-cell landscape in the GI tract. Metabolites produced by commensal microbes, and in particular short-chain fatty acids (SCFAs) including acetate, propionate, and butyrate, also play a critical role in mediating the effect of the gut microbiota on Treg induction both in the intestine and the colon (128–130). While SCFAs levels have been correlated with the number of colonic Tregs, butyrate in particular can support Treg differentiation both *in vitro* and *in vivo* (129). Such effects are not confined to the GI tract, and both butyrate and propionate have been reported to influence peripheral Treg development (128).

Several mechanisms potentially involved in SCFAs regulation of T-cell differentiation have been suggested, including the control of cellular metabolism or G-protein-coupled receptor (GPCR) signaling (131). Indeed, through their integration in the Krebs cycle as Acetyl-CoA, SCFAs can directly affect the cellular energy status and the ATP/ADP levels. This in turn leads to the activation of the mammalian target of rapamycin (mTOR), a critical kinase involved in T-cell differentiation, and can facilitate T effector or Treg differentiation (121, 132). In addition, both butyrate and propionate have been reported to inhibit *de novo* fatty acid synthesis though the deactivation of acetyl-CoA carboxylase 1 (133), a process known to limit Th17 cell differentiation and promote Treg development (134). GPCRs, some of which act as receptors for various SCFAs including acetate, butyrate, propionate, or for niacin, have been linked to the regulation of inflammation in various diseases (135), and GPCR signaling plays a critical role in the regulation of Th17 cell and Treg development (131). Indeed, propionate has been reported to increase colonic Treg numbers, an effect thought to be GPR43 dependent (130). Proprionate also increases GPR15 levels, a homing receptor on colonic Tregs known to contribute to limiting inflammation (130, 136). Both butyrate and niacin have been shown to exert their anti-inflammatory effect by regulating colonic macrophages and dendritic cells in a GPR109A-dependent manner (135). In this report, colonic macrophages and dendritic cells from Niacr1^−/−^ (the gene encoding for GPR109A) mice failed to promote the differentiation of naïve CD4^+^ T-cells into Tregs and IL-10-producing T-cells. However, the effect of SCFAs on T-cell differentiation has been recently reported to be GPR41 and GPR43 independent, suggesting that additional mechanisms might play a role in that process (137).

## Epigenetic Regulation of the Th17/Treg Balance by the Gut Microbiota

**Figure 2:**
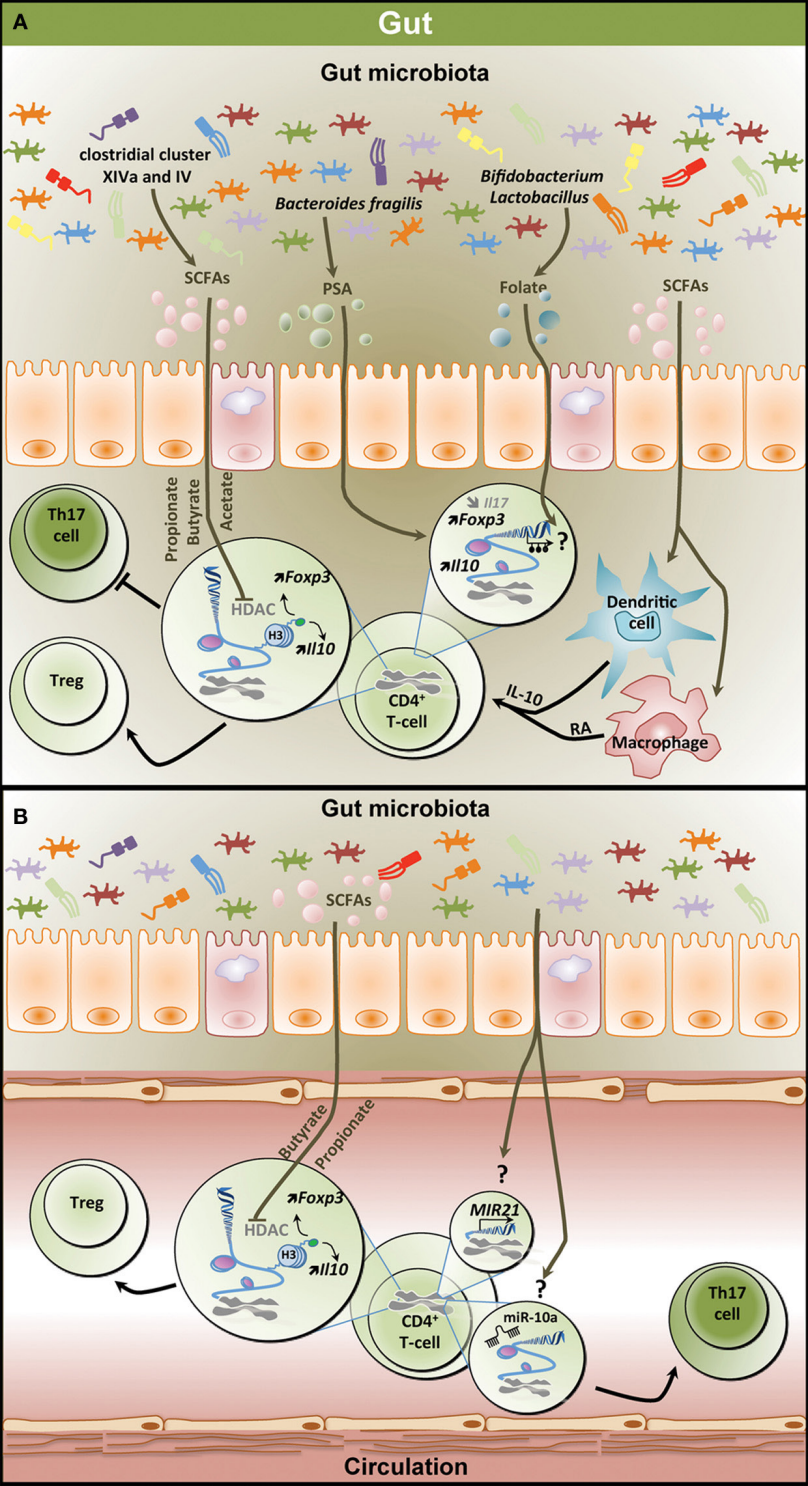
**Epigenetic regulation of the T helper 17/regulatory T cell (Th17/Treg) balance by the gut microbiota**. **(A)** Metabolites derived from the gut microbiota affect the T-cell epigenome influencing the Th17/Treg balance in the gastrointestinal (GI) tract. SCFAs including propionate, butyrate, and acetate which are mostly produced by clostridial clusters VIV and IV exert HDAC inhibitory activity, thereby increasing *Foxp3* and *Il10* expression and promoting Treg differentiation and function. SCFAs can also affect dendritic cells and macrophages, thereby inducing IL-10 and RA production and promoting Treg differentiation. PSA derived from *Bacteroides fragilis* contributes to increased expression of *Il10* and *Foxp3* in T-cell and reduces *Il17* expression. Both *Bifidobacterium* and *Lactobacillus* contribute to the production of the methyl donor folate potentially influencing the methylome in T-cells. **(B)** Effect of microbiota-derived metabolites on the epigenome of peripheral T-cells. Similar to the GI tract, SCFAs increase *Foxp3* and *Il10* expression in peripheral T-cells through HDAC inhibition favoring Treg differentiation. Through unknown mechanisms, the gut microbiota increases the expression of *MIR21* and the levels of miR-10a, potentially promoting Th17 differentiation. SCFAs, short-chain fatty acids; PSA, polysaccharide A; HDAC, histone deacetylase; H3, histone 3; *Foxp3*, forkhead box P3; *Il10*, interleukin 10; RA, retinoic acid; Treg, regulatory T cell.

The inhibitory action of SCFAs on histone deacetylase (HDAC) activity suggests that the gut microbiota could contribute to shaping the host epigenome. This is supported by the recently emerging evidence linking dysbiosis to epigenetic changes in the GI tract, the adipose tissue, or the liver. Such changes have even been suggested to play a role in the development of pathological conditions including obesity and GI cancer (8, 138). The effect of SCFAs on T-cell differentiation could thus be mediated by reshaping the T-cell epigenome (Figure 2). In agreement with this, the effect of butyrate on Treg differentiation is thought to be linked to increased histone H3 acetylation in the *Foxp3* locus (129). Indeed, increased peripheral Treg differentiation in response to butyrate is mediated through increased acetylation of histone 3 at lysine 27 in the *Foxp3* locus (128). In the same study, propionate potentiated peripheral Treg generation through the same HDAC inhibitory effect, while acetate failed to produce such action. The effect of propionate on HDAC inhibition has since been shown to specifically affect HDAC6 and HDAC9 and to be GPR43 dependent (130). Propionate increased not only the frequency and number of colonic Tregs but also their suppressive capacity, potentially through the increased expression of *Foxp3* and *Il10*, and reduced intestinal inflammation. However, it must be noted that both GPR41 and GPR43 have been recently suggested to be dispensable for the inhibition of HDACs by SCFAs in T-cells (137). In this particular study, acetate, propionate, and butyrate were shown to present similar HDAC inhibitory effects and to regulate the mTOR pathway, affecting T-cell differentiation in a cytokine milieu-dependent manner. Although the exact role of GPCRs in SCFA-induced HDAC inhibition still remains to be clarified, there is no doubt that SCFAs play a key role in T-cell differentiation by altering the T-cell epigenome. Several inflammatory diseases have been linked to epigenetic changes that could affect the Th17/Treg balance. In a murine model of colitis, the methyltransferase G9A has been identified as playing a key role in T-cell differentiation, in particular by facilitating dimethylation of histone H3 at lysine 9, supporting Th17 cell and Treg differentiation both *in vivo* and *in vitro* (18). A significant hypomethylation on *MIR21* and increased expression of *MIR21* mRNA has recently been reported in circulating leukocytes from patients diagnosed with CD (139). This could have important consequences on the T-cell profile in these patients as miR-21 has been linked to Th17 cell differentiation (64, 79). The gut microbiota has been shown to downregulate miR-10a in the intestine and dendritic cells in specific pathogen-free mice (140). In this model, miR-10a downregulation was associated with increased expression of IL-12/IL23p40 in dendritic cells. These changes could affect the regulation of intestinal inflammation, as IL-12/IL23p40 play a key role in Th1/Th17 cell differentiation (141). One of the potential mechanisms by which commensal bacteria could influence miRNA expression is through SCFAs. Butyrate has been reported to affect the expression of 44 different miRNAs in a human colon cancer cell line, and in particular members of the miR-106b family (142). These miRNAs included miR-17, a member of the miR-17-92 cluster which is known to promote Th17-mediated inflammation (143). The same miR cluster has further been reported to be critical for the differentiation of Treg, with the loss of miR-17-92 significantly affecting the accumulation of activated Tregs and their differentiation into IL-10-producing cells (76).

## Future Perspectives and Conclusion

Changes in the gut microbiota are intimately linked to significant alterations in Th17/Treg balance, potentially mediated by epigenetic mechanisms, and contributing to the development and establishment of IBD, obesity, and T2D, and possibly other chronic inflammatory conditions. Targeting such epigenetic changes through the use of HDAC inhibitors and miRNA-based therapies has recently started to emerge as a novel therapeutic option in the context of inflammatory diseases (144). This raises the question whether manipulating the gut microbiota could provide a novel approach to reshape the T-cell epigenome and influence the Th17/Treg balance in inflammatory diseases (Figure 2B). Several dietary interventions targeting the gut microbiome have reported positive outcomes in both animal models and human cohorts in IBD, obesity, or T2D (114, 116, 117, 122, 145). Perhaps, some of the most promising dietary interventions include the use of prebiotics and probiotics. Prebiotics are defined as fermented ingredients capable of stimulating bacterial growth and activity in the GI microbiota providing health benefits to the host and include oligosaccharides that are non-digestible, poorly digested carbohydrates and dietary fibers. Supplementation with prebiotics can increase the representation of butyrogenic strains in the gut microbiota, thus supporting an increased production of SCFAs. Increased levels of SCFAs could remodel the T-cell epigenome, favoring Treg differentiation (128–130), in line with the anti-inflammatory effect of SCFAs (129, 130). Even direct oral administration of SCFAs, and in particular butyrate, successfully remodeled the T-cell profile and improved inflammation in mice models of colitis (129, 146). Probiotics are live organisms which, when administered to the host, provide beneficial health effects. They represent an alternative option to prebiotics in modulating the gut microbiota and leading to positive immune adaptations in inflammatory diseases (116, 117). Several microorganisms presenting beneficial immunomodulatory properties that could help protect the host and provide novel therapeutic options for chronic inflammatory disorders have been identified (125, 147). *B. fragilis*, through the expression of PSA, is critical for the suppression of IL-17 production and the protection against inflammation (125). *Bifidobacterium* alone or in combination with prebiotics favors the production of the methyl donor folate (148), which may contribute to Treg survival through epigenetic mechanisms (149), in addition to its well-documented role in health and diseases prevention (150, 151). Similarly, probiotics (*Bifidobacterium* and *Lactobacillus* species) contained in maternal milk have even been suggested to mediate some of the effects of breastfeeding on Treg differentiation (152). These observations highlight the importance of the specific components of the gut microbiota in the establishment and the maintenance of immune homeostasis. Alternatively, untested combinations of bacterial species may produce maximized responses.

Although there is still limited information on the direct effect of microbiota-based therapies on epigenetic regulation of the Th17/Treg balance, the continuously growing literature focusing on such interventions should soon provide the much needed evidence to guide future therapeutic strategies against the inflammatory component of IBD, obesity, or T2D. The systematic investigation of the effect of prebiotics or probiotics on the T-cell epigenome could provide invaluable information on their potential as immunomodulatory therapeutics and support the refining of such interventions. Modulation of the epigenome at gene specific loci, for example, using methyltransferases or acetyltransferases fused to a nuclease-null CRISPR-Cas9 could be used to improve the specificity of such therapies. In the face of the ever-growing pandemic of inflammatory diseases, we are indeed in urgent need of innovative strategies that could specifically target the immune system while also affecting additional contributors to altered immune homeostasis. Challenges that still remain to be overcome include the identification of prebiotics, probiotics, or related metabolites with the highest potential to promote a balanced immune system and the best modalities of administration, as well as the translation of such findings in the context of human diseases. Achieving these outcomes will undoubtedly place us in a better position to tackle inflammatory diseases.

## Author Contributions

AL, SL, RB, LH, MG, and DS contributed to the writing, review, and approval of this manuscript.

## Conflict of Interest Statement

The authors declare that the research was conducted in the absence of any commercial or financial relationships that could be construed as a potential conflict of interest.
